# The Impact of Inflammatory Immune Reactions of the Vascular Niche on Organ Fibrosis

**DOI:** 10.3389/fphar.2021.750509

**Published:** 2021-10-29

**Authors:** Hong-Yan Zhou, Hua Sui, Yang-Jianing Zhao, Hong-Jie Qian, Nan Yang, Lu Liu, Qing Guan, Yue Zhou, Hong-Li Lin, Da-Peng Wang

**Affiliations:** ^1^ The First Affiliated Hospital, Dalian Medical University, Dalian, China; ^2^ Institude college of Integrative Medicine, Dalian Medical University, Dalian, China; ^3^ Department of Nephrology, The First Affiliated Hospital, Dalian Medical University, Dalian, China

**Keywords:** fibrosis, inflammation, vascular niche, endothelial cells, pericytes, macrophages, antifibrosis

## Abstract

Inflammation is a type of defense response against tissue damage, and can be mediated by lymphocytes and macrophages. Fibrosis is induced by tissue injury and inflammation, which leads to an increase in fibrous connective tissue in organs and a decrease in organ parenchyma cells, finally leading to organ dysfunction or even failure. The vascular niche is composed of endothelial cells, pericytes, macrophages, and hematopoietic stem cells. It forms a guiding microenvironment for the behavior of adjacent cells, and mainly exists in the microcirculation, including capillaries. When an organ is damaged, the vascular niche regulates inflammation and affects the repair of organ damage in a variety of ways, such as via its angiocrine function and transformation of myofibroblasts. In this paper, the main roles of vascular niche in the process of organ fibrosis and its mechanism of promoting the progress of fibrosis through inflammatory immunoregulation are summarized. It was proposed that the vascular niche should be regarded as a new therapeutic target for organ fibrosis, suggesting that antifibrotic effects could be achieved by regulating macrophages, inhibiting endothelial-mesenchymal transition, interfering with the angiocrine function of endothelial cells, and inhibiting the transformation of pericytes into myofibroblasts, thus providing new ideas for antifibrosis drug research.

## Introduction

Organ fibrosis comprises an acute or chronic pathological change to organs caused by infection, inflammation, autoimmune reaction, and other factors. Fibrosis causes destruction and reduction of parenchymal cells of organs, activation of fibroblasts, production of the extracellular matrix (ECM) (such as collagen, glycoproteins and adhesion proteins), and continuous deposition of ECM on the basement membrane. This results in continuous progress of organ structural disruption, functional decline, and even exhaustion, which seriously threatens human health and life, and is a hot topic in medical research (Budi et al., 2021). Inflammation is a defense response against tissue damage, and can be mediated by lymphocytes and macrophages. The vascular niche is composed of endothelial cells (ECs), pericytes, macrophages, hematopoietic stem cells, adipocytes and nerve cells, and it forms a guiding microenvironment for the behavior of adjacent cells and mainly exists in the microcirculation, including capillaries (Ribatti et al., 2021; Wermuth et al., 2016). The vascular niche can maintain the steady state, and the normal growth and differentiation of cells by the secretion of angiocrine factors, including transforming growth factor β (TGFβ), vascular endothelial growth factor (VEGF), fibroblast growth factor (FGF), hepatocyte growth factor (HGF), interleukin (IL), platelet-derived growth factor BB (PDGF-BB), EC-derived stem cell factor (SCF) and endothelin (ET) (Rafii et al., 2016; Ribatti et al., 2021; Wermuth et al., 2016).

**FIGURE 1:**
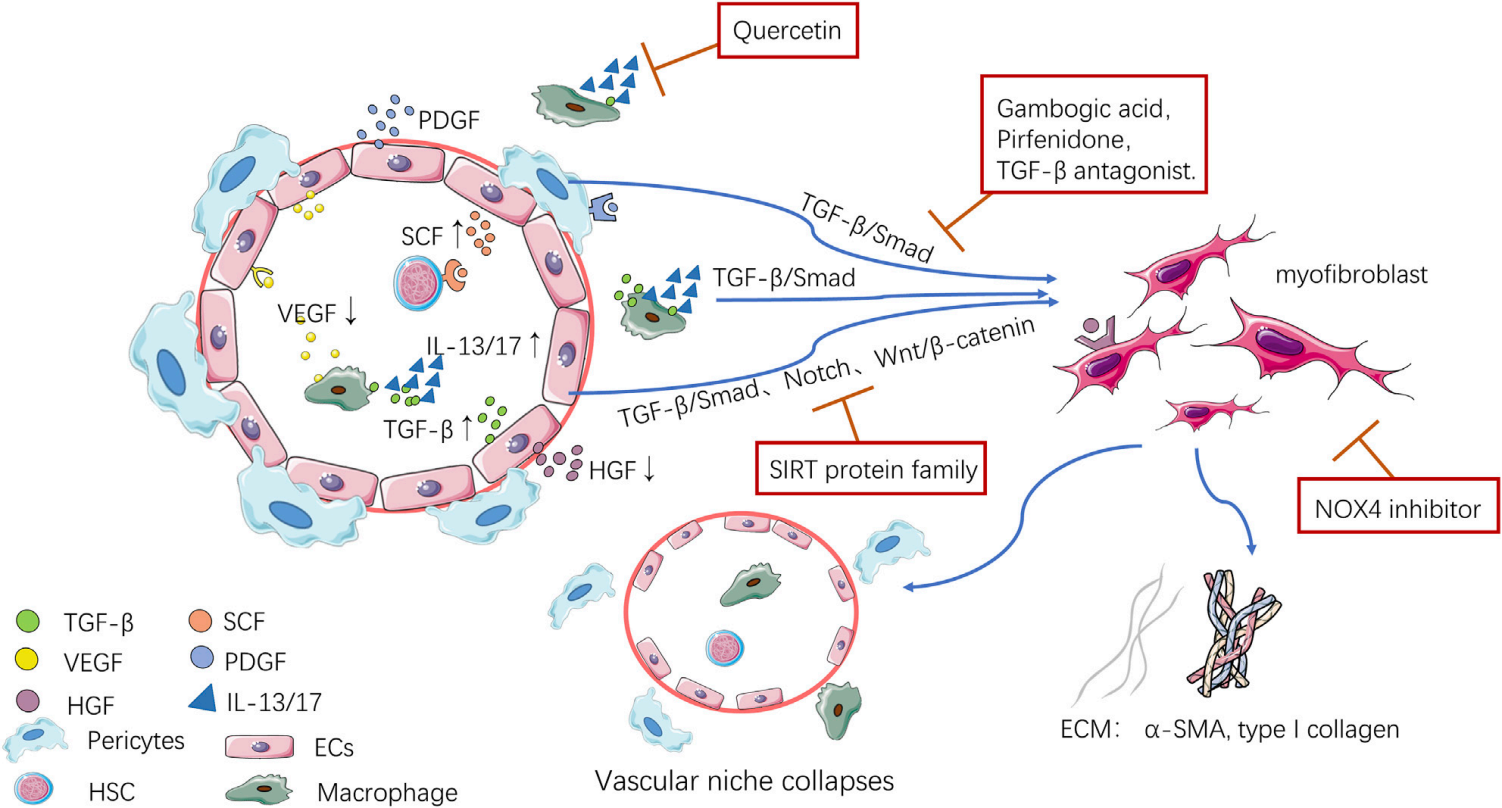
Pathological changes to the vascular niche.

Recently, many studies have shown that the vascular niche plays an important regulatory role in the repair process after organ injury, inflammation and fibrosis. When organs are injured, the vascular niche environment becomes an environment that is not conducive to tissue cells regeneration, and ECs and pericytes secrete various cytokines and inflammatory factors to transform into myofibroblasts, and macrophages express inflammatory factors and pro-fibrosis mediators, which together initiate fibrosis and promote the deposition of ECM (Figure 1). This article summarized the main role of the vascular niche during organ fibrosis and the mechanism by which the vascular niche promotes fibrosis through inflammatory immune regulation at home and abroad in recent years. It was proposed that the vascular niche should be regarded as a new therapeutic target for organ fibrosis. This suggested that antifibrotic effects could be achieved by regulating macrophages, inhibiting endothelial-mesenchymal transition (EndoMT), interfering with the paracrine function of ECs, and inhibiting the transformation of pericytes into myofibroblasts, thus providing new ideas for antifibrosis drug research.

## Role of the Main Cells in the Vascular Niche in Organ Fibrosis

### Endothelial Cells

Endothelial cells (ECs) and pericytes, the most important structural cells in the vascular niche, constitute the microvascular wall. The study of ECs and pericytes further revealed the mechanism of the vascular niche. Genetic and biochemical studies showed that ECs play an important role in maintaining metabolism and organ regeneration. Organ-specific ECs perform many complex tasks by providing a variety of cell stimulating and inhibiting factors, the ECM, and cell chemotactic factors, including VEGF, FGF, IL, TGF-β, PDGF-BB, and ET (Rafii et al., 2016). In fibrotic organs, transplantation of parenchymal stem cells can be used as a treatment; however, the abnormal vascular niche environment of damaged organs usually affects such transplantation. After discovering the angiocrine function of ECs, Cao et al. induced ECs and promoted the synergistic effect of the vascular niche and transplanted stem cells by upregulating the expression of angiocrine factor hepatocyte growth factor (HGF), which successfully stimulated effective organ repair (Cao et al., 2017). In addition, Wermuth et al. found that ET-1 secreted by ECs can not only regulate cardiovascular function, but also promote fibrosis, thereby stimulating the EndoMT induced by TGF-β1 and promoting the expression of mesenchymal fibrosis-associated genes and proteins (Wermuth et al., 2016). Moreover, after the ET-1 gene was knocked out, the expression of ET-1 was downregulated, the number of myofibroblasts was reduced, and the degree of fibrosis was weakened (Arfian et al., 2020).

In addition to indirectly affecting the process of organ fibrosis through angiocrine pathways, Ecs can also participate directly in the progression of fibrosis through EndoMT. EndoMT is a newly discovered type of cell transdifferentiation, which can be induced by TGF-β. It is another important source of myofibroblasts in the process of organ fibrosis. In this process, Ecs lose their endothelial-specific markers and acquire a mesenchymal cell or myofibroblast phenotype, producing excessive amounts ECM components, such as α smooth muscle actin (α-SMA) and type I collagen (Piera-Velazquez et al., 2011). The Sirtuin (SIRT) protein family is related to almost all types of organ fibrosis, and a recent study found that the mechanism by which SIRT improves fibrosis might be related to inhibition of EndoMT (Mazumder et al., 2020). In addition to inhibiting the classical fibrosis pathway to achieve an antifibrotic effect, SIRT can also inhibit the transdifferentiation of Ecs into mesenchymal cells, which can alleviate the renal fibrosis induced by Ang II after activation. Meanwhile *SIRT3* and *SIRT7* gene knockout aggravated the increase in EndoMT and reactive oxygen species, thus causing fibrin deposition (Lin et al., 2018; Liu J. et al., 2019; Wyman et al., 2020).

On the basis of these observations, organ-specific capillary Ecs are considered as special niche cells. On the one hand, they express angiocrine factors, such as pro-fibrosis mediators, pro-inflammatory mediators, and vasoactive substances, to regulate the inflammatory environment of the vascular niche, thus affecting the activation and transformation of various cells in the vascular niche and indirectly participating in the fibrotic process (Rafii et al., 2016). On the other hand, Ecs can increase the source of myofibroblasts in organs through EndoMT, promote deposition of the ECM, and directly participate in fibrosis, when an organ is damaged (Piera-Velazquez et al., 2011). Therefore, we suggest that measures can be taken to intervene with the angiocrine function of Ecs, such as altering in the production of VEGF, TGF-β, and ET-1, or inhibiting the EndoMT of Ecs, which might become a new method to treat fibrosis.

### Pericytes

Pericytes also play an important role in the process of organ fibrosis. Pericytes cover all blood vessels, surround Ecs in capillaries and microvessels, support the blood vessel wall, and express adhesion molecules (e.g., melanoma cell adhesion molecule (MCAM or CD146), platelet derived growth factor receptor beta (PDGFRβ), and chondroitin sulfate proteoglycan 4 (CSPG4 or NG2), playing an important role in the stability and functional regulation of blood vessels (Zhang ZS. et al., 2020). There has also been a breakthrough in our understanding of pericytes in the vascular niche. Pericytes are not just supportive cells around blood vessels, Birbrair et al. identified two kinds of pericytes from skeletal muscle: Type 1 pericytes, which produce adipocytes and fibroblasts, and participate in the progress of fibrosis; and type 2 pericytes, which promote the regeneration of blood vessels and muscles, and might be induced to differentiate into a neural lineage under special conditions (Birbrair et al., 2013; Birbrair et al., 2014). This discovery promoted the development of tissue repair and regenerative medicine. In addition, *in vivo* and *in vitro* studies showed that pericytes can be used as a source of most mesenchymal stem cells (MSCs), and some scholars even suggested renaming MSCs as pericytes. Meanwhile, because of the heterogeneity of MSCs, they can differentiate into chondrocytes under the guidance of TGF-β, and can play an important role in supporting hematopoiesis, organ regeneration, and angiogenesis (Crisan et al., 2012; Caplan, 2017; Joensuu et al., 2018; Kunisaki, 2019).

Kramann et al. verified that perivascular Gli1+ MSC-like cells (i.e., pericytes) are one of the main sources of myofibroblasts in various organs by tracing their genetic lineage (Kramann et al., 2015). Based on that study, Mayr et al. discovered a new pericyte state related to interstitial lung disease (ILD), namely, SSTR2+/CFHR1+ pericytes (somatostatin receptor two and complement factor H related 1) with high disease specificity, which have a pro-inflammatory phenotype and can express various chemokines (Mayr et al., 2021). It has been proven that pericytes separate from Ecs during chronic kidney injury and many studies have found that the separated pericytes show a tendency to differentiate into myofibroblasts (Bábíčková et al., 2017; Liu et al., 2018; Yamaguchi et al., 2020). Perry et al. proved that pericyte expression of 5′-nucleotidase Ecto (NT5E or CD73; an enzyme that can inhibit inflammation) can inhibit the transformation of pericytes into myofibroblasts and attenuate macrophages infiltration (Perry et al., 2019). In ECs, the SIRT protein family can inhibit EndoMT to inhibit fibrosis and it has been proven that SIRT protein family has similar effects in pericytes (Liu ZH. et al., 2019; Su et al., 2020). *SIRT3* gene knockout promoted the transformation from pericytes to myofibroblasts in the heart and kidney, which resulted in upregulation of NADPH oxidase levels and ROS expression in the heart, and decreased glucose metabolism and increase ROS formation in the kidney, leading to dysfunction of the renal endothelium and promotion of fibrosis (Feng et al., 2020; Su et al., 2020).

Unlike the angiocrine function of Ecs, the main role of pericytes in organ fibrosis is to provide a source of myofibroblasts. Pericytes differentiate into myofibroblasts under the induction of TGF-β, and express α-SMA and type I collagen (Yamaguchi et al., 2020). Moreover, inhibiting the transformation of pericytes into myofibroblasts represents an antifibrotic method, including inhibiting the aggregation and activation of pericytes, and separating and transforming them from Ecs. Currently, the pathways known to be involved in this process are the TGF-β, VEGFR, and PDGFR pathways (Liu et al., 2018). Wang et al. successfully proved the feasibility of inhibiting the PDGFR pathway to inhibit organ fibrosis. They used a NOTCH1 inhibitor in a mouse model of pulmonary fibrosis, which successfully inhibited the PDGFRβ/Rho associated coiled-coil containing protein kinase 1 (ROCK1) pathway, inhibited the proliferation and differentiation of pericytes, and reduced the severity of fibrosis (Wang et al., 2019). The effects of other pathways on inhibiting fibrosis have been confirmed in mouse models; however, their effects in clinical application and related drug research and development require further exploration.

### Interactions Between ECs and Pericytes

Ecs and pericytes, as the main structural cells of the vascular niche, jointly maintaining the structural stability of microvessels and the steady state of the microenvironment of the vascular niche. Determining the communication between them will help us to understand the formation, stability, and remodeling of blood vessels and their influence on organ fibrosis. To date, it has been confirmed that ECs have two effects on pericytes. First, they could stimulate the maturation, proliferation, and differentiation of pericytes when in contact with pericytes directly. For example, direct endothelial contact could inhibit the activation of PDGFR-β^+^ pericytes (these pericytes are profibrotic) and stimulate outgrowth of pericyte projections to participate in angiogenesis. (Brandt et al., 2019; Wang et al., 2020). Second, ECs inhibit the transformation of pericytes into myofibroblasts, which might be related to the decreased expression of TFG-β and the secretion of exocrine miR-107 by ECs (Brandt et al., 2019; Wang et al., 2021). It is well-known that pericytes affect endothelial permeability; however there is not enough research about the molecular mechanism of how pericytes affect endothelial cells function. Some scholars suggest that pericytes restrict VEGF signal transmission of local endothelial cells by expressing VEGF receptors, thus affecting EC proliferation and angiogenesis. However, whether pericytes affect vascular niche stability and organ fibrosis has not been determined (Eilken et al., 2017).^.^

### Macrophages

Macrophages are important immune cells in the human body, which exist widely in blood and tissues, and can differentiate into different subgroups according to their different locations and functions, participating in every stage of tissue injury repair (Smigiel and Parks, 2018). The study of different macrophages subsets and their roles in different stages of inflammation and fibrosis will help to identify more antifibrotic therapeutic targets. Here, we mainly discuss the macrophage population in the vascular niche, including tissue-resident macrophages and the circulating mononuclear/macrophage system. Macrophages resident in lung tissue, and include alveolar macrophages and interstitial macrophages. Chakarov et al. performed single cell mRNA sequencing and found that macrophages in the vascular niche are mainly LYVE1^hi^MHCII^lo^ (lymphatic vessel endothelial hyaluronan receptor one and major histocompatibility complex, class II) interstitial macrophages, and express genes related to damage repair and fibrosis at a high level, including *CXCL4* and *TGFβ2* (Chakarov et al., 2019). Macrophages with this phenotype also exist in the vascular niche of other organs; however, whether there are other macrophage populations in the vascular niche remains to be studied. In addition to the expression of TGF-β to initiate fibrosis, there are another study found that M2 macrophages can also transform into myofibroblasts during renal fibrosis, and the more transformed cells, the more severe is the renal interstitial fibrosis (Wang YY. et al., 2017). Zhang et al. found that the liver is rich in the long noncoding RNA (lncRNA) *LFAR1*, and found that the activation of the pro-inflammatory phenotype of macrophages was inhibited after knocking out its gene, which might become a new way to intervene in the inflammatory reaction in the process of liver fibrosis (Zhang K. et al., 2020).

Macrophages living in the vascular niche are also regulated by ECs and pericytes. ECs and pericytes promote the recruitment and maturation of macrophages in the vascular niche by secreting various chemokines and cytokines (such as the transcription factor Nr4a1 (Nur77), intracellular adhesion molecule-1 (Icam-1)) and macrophages can also secrete VEGFA to promote endothelial proliferation (Frangogiannis, 2019; Hernandez and Iruela-Arispe, 2020; Shibahara et al., 2020).

## Inflammation and Fibrosis

An inflammatory reaction occurs after any tissue injury, including infection, trauma, and the actions of immune factors. If the injury range is large, the inflammatory reaction continues to increase and the regeneration ability of the normal parenchymal cells of the organs is inhibited, leading to the initiation of pathological repair, and fibrous connective tissue in the interstitial tissue will proliferate, leading to fibrosis. T-lymphocytes, monocytes, macrophages, and neutrophils play an important role in the process of inflammation. These cells release cytokines (e.g., TGF-β, PDGF, IL-13, and IL-17) and chemokines (e.g., CXCL12, CCL2, and CCL18) to achieve the functions of promoting fibrosis and resisting fibrosis (Kolahian et al., 2016; Eming et al., 2017; Mack, 2018). Research on inflammation in the process of pulmonary fibrosis is very mature, and drugs (such as Pirfenidone and nintedanib) can inhibit inflammatory cell infiltration and the expression of pro-inflammatory mediators (Kolahian et al., 2016). TGF-β has been proven to be the key mediator of fibrosis in various organs. Lan et al. found that Smads, the downstream signal factors of TGF-β, also has a certain regulatory effect on kidney inflammation, and different Smad molecules have different roles in kidney fibrosis and inflammation (Lan, 2011). In the process of myocardial fibrosis, fibroblasts not only produce the ECM, but also express SRY-box transcription factor 9 (SOX9), which is a main regulator of inflammation and functions to increase leukocyte infiltration and the expression of inflammatory mediators (CXCL13 and IL-6) in myocardial tissue, suggesting SOX9 as a new therapeutic target during myocardial infarction (Scharf et al., 2019). The liver, as an immune organ, shows a more obvious inflammatory reaction in the process of injury, in which innate immunity and acquired immunity interact to cause the occurrence of an inflammatory reaction. At the same time, macrophages in the vascular niche express TGF-β1, which induces hepatic stellate cells to differentiate into myofibroblasts and thus initiate liver fibrosis (Koyama and Brenner, 2017; Sutti and Albano, 2020). All these findings indicate that persistent inflammation is closely related to the occurrence and development of organ fibrosis, and macrophages in the vascular niche might be an important therapeutic target to regulate organ fibrosis through the vascular niche.

## Relationship Between the Vascular Niche and Fibrosis of Various Organs

### The Vascular Niche and Pulmonary Fibrosis

**FIGURE 2:**
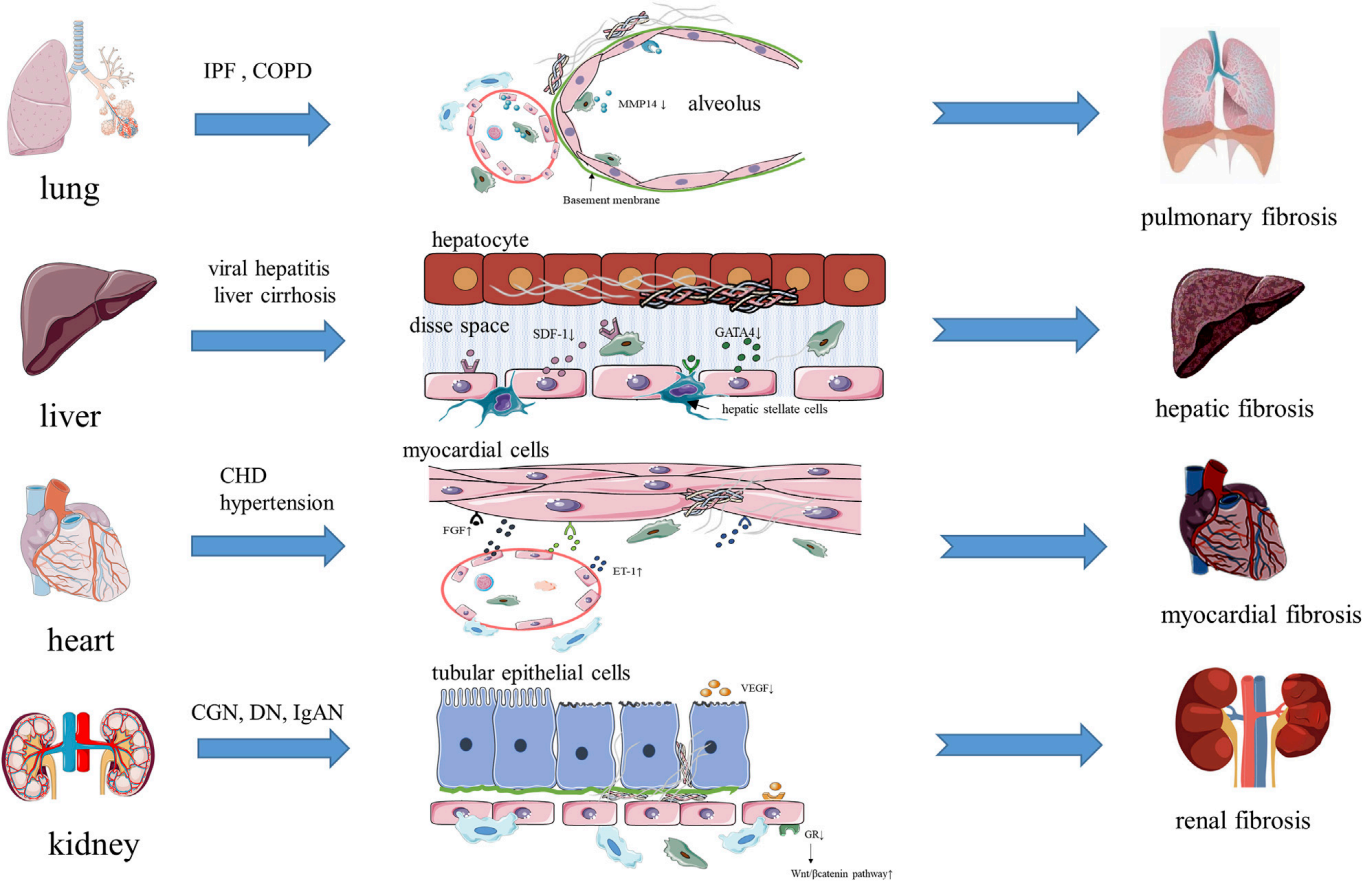
The vascular niche and organ fibrosis.

Pulmonary fibrosis means that the regeneration ability of alveoli is inhibited after long-term chronic injury (such as idiopathic pulmonary fibrosis (IPF), chronic obstructive pulmonary disease (COPD), asthma), and biomarkers with diagnostic specificity (such as matrix metalloproteinase 7 (MMP7) and Krebs Von Den Lungen-6 (KL-6, also known as mucin 1)) are expressed. In addition, fibrin deposits replace normal alveolar tissue, resulting in irreversible fibrosis (Han et al., 2018; Drakopanagiotakis et al., 2018) (Figure 2). Alveoli are rich in capillaries, and pulmonary capillary endothelial cells (PCECs), as the main cells of the vascular niche, are embedded between type 1 alveolar cells and are regulated by the angiocrine factor VEGFA expressed by type 1 alveolar cells, thereby promoting local alveolar angiogenesis (Vila Ellis et al., 2020). Therefore, the steady state of the vascular niche is very important for the structure and regeneration ability of lung tissue. Ding et al. found that MMP14, produced by PCECs and macrophages, could regulates alveolar formation by stimulating epithelial proliferation and migration (Ding et al., 2011). They also found that in PCECs with chronic injury, the expression of chemokine (C-X-C motif) receptor 7 (CXCR7) was inhibited, perivascular fibroblasts continued to be activated, and macrophages interacted with PCECs to form inflammatory vascular niche and stimulate pulmonary fibrosis, while infusing *sdf1+/+* (SDF1, stromal cell-derived factor 1) platelets into a left lung unilateral pneumonectomy (PNX) model mouse can increases the expression of CXCR7, induces the MMP14 secretion of PCECs, and form a vascular niche microenvironment that promotes alveolus formation (Rafii et al., 2015; Cao et al., 2016). After these studies, Ding’s team suggested that the vascular niche and its cells should be regarded as a whole, and by analyzing its steady state, they found that the balanced steady state can promote the normal repair of tissue injury, and once the balance of the vascular niche is destroyed, inflammation persists, which can stimulate the abnormal repair of injury and lead to fibrosis (Ding et al., 2011; Rafii et al., 2015; Cao et al., 2016). In addition, Cao et al. found that HGF, expressed by PCECs, can suppress the pro-fibrotic expression of NADPH oxidase 4 (NOX4) in perivascular lung fibroblasts, and the vascular microenvironment of mouse treated with a NOX4 inhibitor could change an epithelially-prohibitive microenvironment (inhibiting the proliferation of epithelial cell) to an epithelially-active microenvironment (promoting epithelial cell regeneration and proliferation), thus realizing the regenerative treatment of fibrosis (Cao et al., 2017). Brody et al. found that CCR2^+^(chemokine C-C motif receptor 2) macrophages accumulated significantly around fibrous tissue while studying lung tissue from patients with IPF, and that the antifibrotic drug pirfenidone could effectively reduce the accumulation of these macrophages (Brody et al., 2021). In addition, the Wnt inhibitor Dickkopf-1 (DKK 1) also had an effect on the vascular niche in chronic lung diseases (including COPD and emphysema), as shown by the reduced expression of the profibrotic phenotype of mesenchymal vascular progenitor cells (MVPCs), inhibition of the TGFβ and PDGF signaling pathways in ECs, and the regulation of pulmonary smooth muscle cell differentiation and proliferation (Summers et al., 2021). All these studies indicated that the steady state of the vascular niche is closely related to alveolar repair and fibrosis in the process of lung injury, and could even be used as a new therapeutic target for pulmonary fibrosis.

### The Vascular Niche and Hepatic Fibrosis

Hepatic fibrosis is a common pathological process in the end stage of various chronic liver injuries (such as hepatitis, fatty liver and drug-induced liver injury). Excessive ECM components, such as glycoproteins and collagen, are deposited in the process of repeated destruction and repair of hepatocytes ((Seki and Brenner, 2015; Koyama and Brenner, 2017) (Figure 2). The activated myofibroblasts that produce the ECM in liver mainly come from hepatic stellate cells (i.e., pericytes of the hepatic vascular niche), and TGF-β and PDGF are two main cytokines that promote the activation and proliferation of hepatic stellate cells, which are mainly produced by macrophages gathered in circulation and macrophages in the vascular niche (Seki and Brenner, 2015; Koyama and Brenner, 2017; Trivedi et al., 2021). The hepatic vascular niche is mainly composed of hepatic sinusoidal endothelial cells (HSECs). Unlike endothelial cells forming other continuous cell layers, HSECs mainly constitute discontinuous and permeable microvessels, and lack a basement membrane, which can reduce the deposition of the ECM (Géraud et al., 2017). During liver diseases, an immune response is initiated, in which macrophages, HSECs, and hepatic stellate cells are activated, and the expression of SDF-1 increases, which promotes the formation of microvessels in the injured area, activates hematopoietic stem cells, and promotes the normal repair of tissues, meanwhile the selective activation of SDF-1 receptors CXCR7 on HSECs can promote the secretion of angiocrine factors by HSECs, which can mediate the repair and regeneration of damaged organs (Ding et al., 2014; Liepelt and Tacke, 2016; Shido et al., 2017). In addition, the transcription factor GATA binding protein 4 (GATA4), expressed by HSECs, is the main regulator of liver microvessels, which limits the transformation of HSECs into continuous capillaries (Géraud et al., 2017). Meanwhile, capillarization is characterized by ectopic deposition of the basement membrane and the formation of continuous endothelial cell layer, which is beneficial for ECM deposition and causes the tissue to undergo pathological damage repair (Géraud et al., 2017). Winkler et al. found that GATA4 controls liver fibrosis and regeneration by repressing transcription factor MYC activation and pro-fibrotic angiocrine signaling. They determined the molecular mechanism, whereby the expression of angiocrine factors changed and led to fibrosis in HSECs lacking GATA4. Factors such as platelet derived growth factor subunit B (PDGF-BB), SPARC like 1 (SPARCL1), endothelial cell specific molecule 1 (ESM1) and insulin like growth factor binding protein 5 (IGFBP5), which promote fibrosis, were upregulated; however, bone morphogenetic protein 2 (BMP2), hepatocyte growth factor (HGF), Wnt family member 2 (WNT2), which promote liver regeneration, were downregulated, and hepatic stellate cells were activated, which jointly promoted the development of fibrosis in mouse and human livers (Winkler et al., 2021). Duan’ study revealed another way of promoting fibrosis in HSECs. Notch signaling in highly differentiated HSECs is activated when hepatocytes are damaged, which downregulates endothelial nitric oxide synthase (eNOS)-soluble guanylyl cyclase (sGC) signal transduction, reduces the expression of WNT2a, 9b, and HGF, and results in the discontinuous endothelial cell layer of hepatic sinus capillaries dedifferentiating into a continuous endothelial cell layer, meanwhile the activation of hepatic stellate cells and the production and deposition of the ECM increase, which affects hepatocyte proliferation and liver regeneration (Duan et al., 2018). Ramachandran et al. also found a new fibrosis-associated TREM2^+^ CD9^+^ macrophage subpopulation in patients with liver cirrhosis, which are located in the vascular niche and promote hepatic fibrosis by promoting the expression of collagen in hepatic stellate cells (Ramachandran et al., 2019). Changes to the above-mentioned signal pathways result from abnormal activation of HSECs, which leads to an imbalance of the vascular niche and angiocrine factors, and activation of macrophages and the fibrosis pathway. These studies suggest that the steady state of the vascular niche is closely related to the repair and fibrosis of liver tissue during liver injury, which also provides a new idea for the targeted treatment of liver fibrosis.

### The Vascular Niche and Myocardial Fibrosis

Myocardial fibrosis is a process in which activated fibroblasts produce ECM, and macrophages and mast cells produce fibrogenic mediators when myocardial cells are injured (such as in CHD (coronary atherosclerotic heart disease) and hypertension) (Kong et al., 2014) (Figure 2). In cardiovascular diseases, the hematopoietic supply is very important to cardiomyocytes, while hematopoietic stem cells (HSCs) are mainly located in the vascular niche around the blood sinuses (a type of capillary vessel). ECs, as the main effector cells in the cardiac vascular niche, produce a variety of factors to regulate HSC function, such as EC-derived stem cell factor (SCF), and SCF can affect the proliferation and differentiation of HSCs and interfere with the progress of myocardial fibrosis (Xu et al., 2018; Poller et al., 2020). Like ECs, pericytes can also support the growth of HSCs by expressing regulatory factors to intervene the injury repair of myocardium and inhibit fibrosis progression (Sá da Bandeira et al., 2017). The steady-state regulation function of the cardiac vascular niche is similar to that of lung and liver. ECs express profibrotic mediators (such as TGF-β1, FGF, ET-1) to directly promote myocardial fibrosis, and also express function regulators of HSCs (SCF) to regulate the growth environment of HSCs. In addition, ECs also express adhesion molecules, pro-inflammatory factors, and chemokines to promote the recruitment of macrophages and lymphocytes, thus aggravating myocardial inflammation and fibrosis (Kong er al. 2014; Frangogiannis, 2019). In contrast to the lung and liver, another function of ECs in the myocardial vascular niche is to provide the source of myofibroblasts and promote the deposition of the ECM through EndoMT. Since EndoMT was first discovered in myocardial cells, many scholars have studied its molecular mechanism, and concluded that the main signaling pathways of EndoMT are TGF-β/SMAD, NOTCH, and WNT/β-catenin (Sniegon et al., 2017; Cheng et al., 2021). Moreover, EndoMT was identified as the cause of fibrosis in the kidney, heart, and liver, which might result in EndoMT becoming a new therapeutic target for preventing, delaying, and treating fibrosis (Cruz-Solbes and Youker, 2017). Li et al. used a constitutive endothelial Tie1–Cre line to trace all ECs in an animal model of ascending aortic constriction, and found that a substantial number of Tie1–Cre labelled cells also expressed the fibroblast marker fibroblast-specific protein 1 (FSP1), indicating that these cells had undergone EndoMT (Li et al., 2018). This study suggested that besides bone marrow-derived fibroblasts, ECs in the vascular niche were another source of myofibroblasts. The findings of the above studies indicated that the vascular niche could maintain the proliferation and differentiation of HSCs and macrophages by regulating the steady state, and can directly promote fibrosis by expressing pro-inflammatory and pro-fibrotic factors. More importantly, the vascular niche can provide myofibroblasts through EndoMT directly to increase the deposition of the ECM. If timely intervention measures are undertaken against these processes, cardiac fibrosis could be inhibited effectively and cardiac function improved.

### The Vascular Niche and Renal Fibrosis

The essence of renal fibrosis is scar accumulation caused by pathological fiber repair in the parenchyma during the progression of diseases such as chronic glomerulonephritis (CGN), IgA nephropathy (IgAN), and diabetic nephropathy (DN), while the excessive deposition of the ECM separates the basement membrane of renal tubules from the capillary around renal tubules, causing renal tubule interstitial fibrosis (Humphreys, 2017) (Figure 2). Myofibroblasts are major matrix secreting cells, while pericytes are one of the major providers of myofibroblasts (Humphreys, 2017; Kuppe et al., 2021). It has been proven that there are changes in the structure and steady state of the vascular niche in all of kidney diseases, such as capillary endothelial damage, separation of pericytes from capillary endothelial cells, capillary rarefaction, the decreased expression of pro-angiogenic factors (VEGF, angiopoietin 1) and anti-angiogenic factors (angiostatin 1), and all of these can accelerate glomerulosclerosis and tubulointerstitial fibrosis (Bábíčková et al., 2017; Tanabe et al., 2020). ECs not only severs as the classical barrier function in many physiological processes in the kidney, they can also participate in regulating vascular tension and inflammatory reactions by secreting vasoactive substances such as ET, tropomyosin 1 (TM-1), Von Willebrand factor (vWF) and inflammatory factors, and maintain the steady state of the vascular niche, providing a good environment for the survival and proliferation of hematopoietic stem cells (Rafii et al., 2016; Yang et al., 2019). In addition, the glucocorticoid receptor (GR) on ECs plays a key role in the process of diabetic renal fibrosis, which can downregulate the classical WNT signaling pathway to inhibit fibrosis, prompting researchers to suggest that endothelial GR is a key anti-EndoMT molecule, which might become an antifibrotic therapeutic target for diabetic renal fibrosis (Srivastava et al., 2021). Similar to the process of myocardial fibrosis, EndoMT also has a certain influence on accelerating renal fibrosis, and it has been proposed that EndoMT, which causes independent endothelial dysfunction, is also worth noting (Yang et al., 2019). In the process of renal fibrosis, macrophages not only act as immune cells, but also directly transform into myofibroblasts in the damaged kidney mediated by the TGFβ1/SMAD3 pathway, via a process known as macrophage-to-myofibroblast transition (MMT) (Tang et al., 2019). During the process of renal fibrosis, pericytes have become the main source of myofibroblasts, and EndoMT and the transformation of pericytes into myofibroblasts both lead to the changes in the normal structure of the vascular niche. Taking measures to intervene the transformation of pericytes into myofibroblasts and reducing the separation of pericytes from ECs might become a new method for treatment of renal fibrosis.

## New Therapeutic Targets of Organ Fibrosis

At present, the methods used to treat fibrosis include blocking the TGF-β/Smad signaling pathway, regulating the SK1/S1P signaling pathway, and antagonizing the vasoactive peptide receptor. The main representative drugs are farnesoid X receptor (FXR) agonists, TGF-β antagonists and Pirfenidone. Among them, nintedanib and pirfenidone, as representative drugs for the treatment of IPF, can slow down the rate of decline of lung function in IPF, and have some effect in reducing the risk of IPF mortality and acute exacerbations, while pamrevlumab has a relatively better effect in reducing the decline of lung function compared with pirfenidone and nintedanib (Di Martino et al., 2021; Petnak et al., 2021). Thus developing more effective antifibrosis drugs or early intervention drugs remains a research hotspot (Ren et al., 2020). We have described the main role of ECs, pericytes, and macrophages in the vascular niche during fibrosis, which provides us with new directions to find drug targets, such as screening for more single or compound drugs to inhibit organ inflammation and fibrosis according to the molecular mechanisms of the vascular niche.

Yue et al. found that the process of fibrosis can be disrupted by inhibiting the capillarization of HSECs. Plumbagin can increase and expand the fenestra of HSECs, thin the extracellular basement membrane, and reduce its scope, thus decreasing the degree of capillarization of HSECs(Peng et al., 2021). Quercetin has a variety of remarkable biological activities. In recent years, quercetin was demonstrated to improve the endothelial dysfunction of pulmonary vessels, antagonize the vasoconstriction and fibrosis induced by ET-1, and effectively inhibit the EndoMT mediated by TGF-β (Huang et al., 2017; Dagher et al., 2021). Other drugs that show antifibrotic activity by intervening in the EndoMT include gambogic acid (in a model of pulmonary fibrosis) and dihydroartemisinin (in a model of skin fibrosis). However, their antifibrotic effects in other organs remain to be studied, and whether there are other drugs that act via novel antifibrotic mechanisms remains to be discovered (Qu et al., 2016; Li et al., 2021). These studies indicated that EndoMT in the vascular niche will become an important antifibrotic therapeutic target in the future.

Based on the above summary of the cellular and molecular mechanisms of the vascular niche during fibrosis, we speculated that fibrosis could also be inhibited by interfering with the angiocrine function of ECs, such as inhibiting the expression of PDGF, VEGF, TGF-β, ET-1, and other molecules. The forkhead box (FOX) family plays an important role in regulating the survival, proliferation, and differentiation of stem cells, and its role in preventing pathological cardiac fibrosis in the heart has also been confirmed (Wang J. et al., 2017). In terms of interfering with the function of ECs in the vascular niche, scholars have found that FOXP1 in ECs inhibits the proliferation and transformation of fibroblasts by downregulating TGF-β1 signaling, while FOXC1 inhibits the expression of inflammatory factors and fibrotic factors by promoting angiogenesis and anti-inflammatory cytokines expression, thus maintaining a microenvironment favorable for the survival of mesenchymal stem cells in the vascular niche, and promoting myocardial repair (Liu J. et al., 2019; Zhao et al., 2020). Inhibiting the transformation of pericytes into myofibroblasts in the vascular niche is a clear intervention method for organ fibrosis. Pericytes play a more significant role in kidney; therefore, this method might have a more significant therapeutic effect on renal fibrosis. Current research shows that intervention in the endothelial-pericyte interaction, such as promoting the separation of pericytes from capillary ECs, can generate and stabilize blood vessels after organ injury; however, whether this has an impact on organ fibrosis is not clear (Perrot et al., 2020).

In addition, Lu et al. found that quercetin could inhibit the infiltration of M1 macrophages into the renal interstitium to reduce inflammation and inhibited the activation of M2 macrophages to reduce the excessive accumulation of the ECM, thus effectively treating renal interstitial fibrosis (Lu et al., 2018). That study demonstrated the feasibility of using vascular niche macrophages as targets for anti-inflammatory and antifibrotic therapy.

Therefore, it is feasible to select the vascular niche as a new target for antifibrotic therapy, which can be achieved by inhibiting the EndoMT, interfering with the angiocrine function of ECs, inhibiting the transformation of pericytes into myofibroblasts, and regulating macrophages.

## Discussion

The vascular niche is a relatively independent entity in organs and tissues. ECs are the core of the vascular niche under physiological conditions, and mediate various signaling pathways to maintain its steady state via autocrine and angiocrine pathways. Under pathological conditions, the vascular niche balance is broken, and ECs secrete various angiocrine, inflammatory, and pro-inflammatory factors, which change the microenvironment and regulate the activation and expression of inflammatory mediators of macrophages in the vascular niche. Meanwhile, during the initiation of EndoMT, pericytes are activated and transformed into myofibroblasts to promote deposition of the ECM, and parenchymal cells are lost, which leads to a disordered vascular niche and promotes the occurrence and development of fibrosis. According to studies on the molecular mechanism of the vascular niche in the process of fibrosis, we proposed new methods to exploit vascular niche as anti-inflammatory and antifibrotic targets, such as regulating macrophages, inhibiting EndoMT, interfering with the angiocrine function of ECs, and inhibiting the transformation of pericytes into myofibroblasts. This will provide new directions for drug research, such as investigating the protective effect of drugs on the vascular niche, clarifying the molecular mechanism of the regulation of key signal pathways, identifying more effective drugs to treat inflammation and fibrosis, and guiding the treatment and rehabilitation of organ fibrosis using modern medicine and Traditional Chinese Medicine.
